# Detection of antibody subclasses IgA, IgM and IgG against HPV L1 in HPV-positive oropharyngeal squamous cell carcinoma patients: a pilot study

**DOI:** 10.1007/s00405-024-08537-9

**Published:** 2024-03-05

**Authors:** Thomas Weiland, Jakob Zgubic, Luka Brcic, Dietmar Thurnher

**Affiliations:** 1 Department of Otorhinolaryngology-Head & Neck Surgery, Medical University of Graz, Auenbruggerplatz 26, 8036 Graz, Austria; 2 Diagnostic and Research Institute of Pathology, Medical University of Graz, Graz, Austria

**Keywords:** HPV-positive oropharyngeal carcinoma, Antibodies, Antibody subclasses, Biomarker, Tumor recurrence

## Abstract

**Purpose:**

Despite prognostic superiority of HPV-positive oropharyngeal squamous cell carcinoma (OPSCC), up to 25% of patients will suffer from recurrence within the first 5 years. Therefore, it is of great scientific interest to find relevant biomarkers to identify patients at risk. In this prospective observational study, we aimed to investigate the dynamics of HPV-L1 capsid protein specific antibody (AB) subclasses IgA, IgM, and IgG in HPV-positive OPSCC patients under therapy.

**Methods:**

Serum samples from HPV-positive OPSCC patients, identified by positive p16-immunohistochemistry, were collected before and during tumor-specific therapy and 3–6 months during follow-up. They were analyzed for the presence of HPV-L1 AB subclasses IgA, IgM, and IgG using an HPV-L1-specific immuno-assay. Additionally, a PCR-based HPV-DNA detection from the tumor tissue was performed.

**Results:**

Altogether, 33 patients with a mean follow-up of 55 months were included. Analysis of a total of 226 serum samples revealed that the most common L1-AB-subclass pattern was characterized by IgG >  > IgA > IgM without significant fluctuation during the course of disease. Patients with excessive IgG levels tended to higher tumor stages and three out of three patients with disease recurrence showed increasing IgG AB titers beforehand. Seven patients showed an IgA dominance at diagnosis, which was associated with a better disease-free survival.

**Conclusion:**

Despite limited cases, our prospective pilot study revealed promising trends in HPV L1 AB subclasses and may contribute useful information for future risk stratification and post-treatment monitoring in HPV-positive OPSCC patients.

**Supplementary Information:**

The online version contains supplementary material available at 10.1007/s00405-024-08537-9.

## Background

With increasing incidences in developed countries, HPV-positive OPSCCs are considered to be more sensitive to chemotherapy, radiation and combined treatment protocols compared to HPV-negative tumors. The p16 protein, routinely determined by immunohistochemistry, is therefore considered to be the most important prognostic marker in this entity [1, 2]. Yet, a recent study by Mehanna et al. showed that there is often a discrepancy between p16 IHC detection and HPV DNA or RNA status. Patients with a positive p16 but negative HPV DNA/RNA status were associated with an inferior prognosis [3]. The existence of a subgroup with poorer prognosis within the prognostically favored group of HPV-positive OPSCCs might explain poorer outcomes in dose de-escalation prospective studies [4–7]. Another hypothesis assumes that certain genetic subtypes of HPV-positive OPSCCs result in different therapy responses [8]. Although there have been promising approaches to tumor markers in HPV-positive OPSCC in the past, such as the determination of ABs against E6 and E7 proteins, but also the detection of circulating HPV tumor DNA (ctDNA) in plasma, study results of these methods have been too heterogeneous and not valid enough to allow usage in clinical routine yet [9–12]. For this reason, it is essential to examine alternative biomarkers for risk stratification to better adapt therapy regimens and for monitoring therapy success. Since it is known that immune responses are important not only concerning the development of tumors but also for prognostic information, an important question is what role the immune system plays in the success of therapy and to what extent the immune response can be mapped [13]. In a previous study, we prospectively examined OPSCC patients with a subtype-specific competitive serological assay based on an HPV16-L1-specific monoclonal antibody before and after therapy. The HPV L1 protein is a structural protein that, together with the L2 protein, forms the capsid of the HPV DNA. The HPV L1 related antibody response was generally seen as a marker of cumulative HPV infection, unsuitable for tumor diagnostics. As part of our previous study, however, we were able to show immunohistochemically that HPV-associated tumors express HPV L1 and we further illustrated that antibody titers in most cases reflect the course of disease [14, 15]. We have also shown in individual cases that an increase in the HPV-L1 antibody titer could indicate a possible recurrence of the disease [14, 15].

In this pilot study, we aimed to investigate the immune response at the level of HPV-L1 antibody subclasses IgA, IgM, and IgG in the context of tumor-specific therapy and follow-up in a cohort of HPV-associated OPSCC patients.

## Methods

### Study population

In this prospective observational study, in total 226 serum samples and clinical data of 33 patients with histologically confirmed OPSCC were collected at the Department of Otorhinolaryngology, Head and Neck Surgery of the Medical University of Graz at diagnosis, under therapy, and during follow-up visits 3–6 months and 6–12 months after completion of therapy (see workflow diagram in the supplements as Supp. 1).

As part of oncological follow-up, regular clinical examinations and radiological imaging were performed every 3 to 6 months according to national guidelines.

The patients had to meet the following criteria for inclusion in the study:

- Immunohistochemically proven p16 positivity from the tumor tissue (p16 Histology KIT, Ventana Roche Diagnostics, Switzerland).
- A curative therapeutic approach according to the tumor board decision.

The following exclusion criteria were defined:

- Patients with recurrent or secondary tumors in the head and neck area.
- Patients in a poor general condition not suitable for any curative tumor-specific therapy.
- Patients with severe cognitive impairment.

Furthermore, study participants were asked about their HPV vaccination status in the context of study enrollment and their medical history was screened for other HPV-induced diseases, like anogenital carcinoma.

In addition to p16 IHC, a PCR-based subtype-specific HPV DNA detection (HPV 3.5 LCD Array KIT, Chipron^®^, Germany) was performed from the histological tumor specimens at initial diagnosis.

### HPV-L1 specific immuno-assay

Serum samples were analyzed for the presence of the HPV-L1-AB subclasses IgA, IgM, and IgG using a commercially available HPV16- and HPV33-specific immuno-assay (Abcerion Diagnostics GmbH, Germany) in concordance with the HPV-PCR subtype detection. In short, serum samples were incubated with HPV L1-specific antigen-captured microtiter plates. After a washing step antibody subclass-specific detection was carried out using horseradish peroxidase (HRP) conjugated anti-IgA, IgM, and IgG antisera. Ready to use tetramethylbenzidine (TMB) was used for color development, and sulfuric acid as a stopping solution. Test results were documented by using a microtiter plate reader, according to the manufacturer’s instructions.

### Statistical analysis

All statistical analysis was performed using IBM SPSS Statistics version 29 (IBM Corp., New York, NY, USA). Shapiro–Wilk-Test has been used to test for normality. Frequencies in nonparametric data are shown with median and range. The Mann–Whitney *U* Test was performed to compare IgG/IgA ratio at diagnosis and gender (male/female), smoking-status (yes/no), alcohol-status (yes/no), and outcome (relapse/no relapse). Furthermore IgG/IgA ratio and outcome (relapse/no relapse) at four timepoints (T0 = at diagnosis, T1 = treatment start, T2 = first follow-up 3–6 months post treatment, and T3 = second follow-up 6–12 months post treatment) were separately tested for significance using Mann–Whitney *U* Test. Pearson’s Chi^2^-test was used to test for association between qualitative variables (AJCC-stages and antibody patterns). Kruskal–Wallis-Test was performed for significance testing in non-parametric data including IgG/IgA ratio at diagnosis, and AJCC-stages. A significance level of α = 0.05 was used. From the AB titers at diagnosis (baseline titers), a definition was added to categorize the different antibody subclasses based on the ratio of the titer levels to one another. The following categories were defined: IgG >  > IgA: > 50%, IgG > IgA: 10–50%, IgG ≈ IgA: ± 10%, IgA > IgG: > 10%, IgG/IgA > IgM: > 10%.

## Results

### Study cohort

We identified 33 cases, 27 male and six female, diagnosed with HPV-positive OPSCC between 2016 to 2021. The median age at diagnosis was 64.7 years (46–89). Tobacco consumption of ≥ 20 pack years (PY) was reported in eleven cases (31.4%), and five patients (14.7%) reported regular alcohol consumption. Only four cases (11.8%) presented with an ECOG score of 1, the remaining cases presented with a score of 0. The tumor was localized in the palatal tonsils (79%) and the base of the tongue (BOT) (15%). Two cases have been diagnosed as CUP. Most cases (27/33, 81.8%) presented with early-stage disease (AJCC staging I and II). Six cases (18%) presented with stage III disease. All cases stained positive for p16. The most frequently identified HPV genotype was HPV16 (88%) followed by HPV33 (6%). PCR for HPV was negative in two cases. The median follow-up time was 55 months. See Table 1 for an overview of patient characteristics.

**Table 1:** Clinical characteristics of study population

	Number of patients
Gender	
Male	27
Female	6
Age^a^	
Median	64.7
Range	46–89
Smoker^b^	
No	22
yes	11
Alcohol^c^	
No	28
Yes	5
ECOG^d^	
0	29
1	4
2–5	0
AJCC-staging^e^	
I	13
II	14
III	6
IV	0
HPV-DNA	
HPV 16	29
HPV 33	2
Negative	2
p16-IHC	
Positive	33
Negative	0
Localisation	
Tonsillar complex	26
Base of the tongue	5
CUP	2

^a^Age at Diagnosis

^b^Yes:  ≥ 20 PY, No: < 20 PY

^c^Regular alcohol consumption ≥ 3 drinks per day

^d^ECOG at diagnosis

^e^AJCC edition 8 staging for p16 pos. cases

### Evaluation of HPV-L1 antibody profiles

**Fig. 1:**
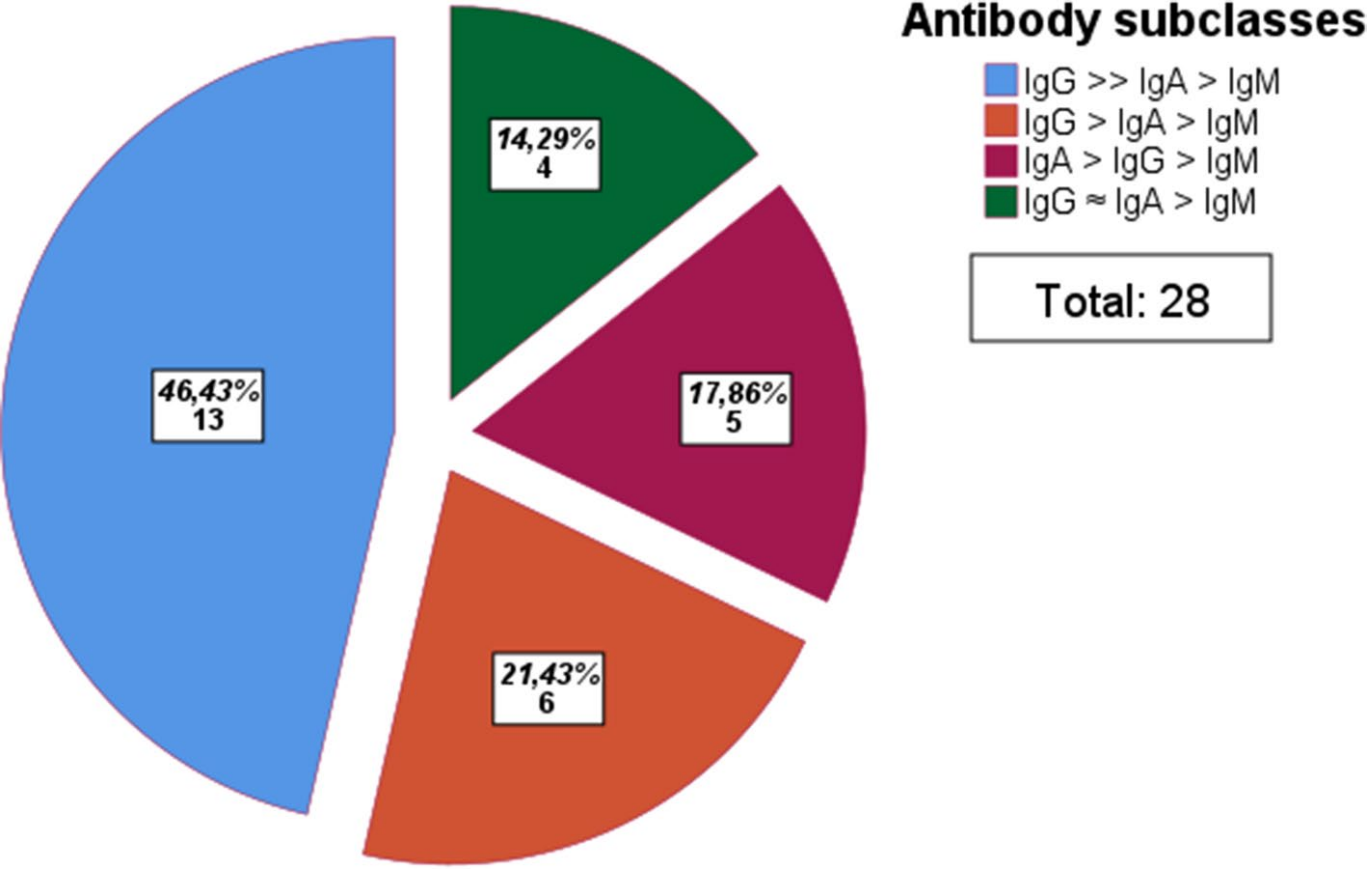
Frequencies of antibody subclasses at diagnosis. Category definition: IgG >  > IgA: > 50%, IgG > IgA: 10–50%, IgG≈IgA: ± 10%, IgA > IgG: > 10%, IgG/IgA > IgM: > 10%. Non-curved numbers represent total numbers, curved numbers represent percentage of total number (*n* = 28). 1st group defined by IgG >  > IgA > IgM, 2nd group defined by IgG > IgA > IgM, 3rd group defined by IgA > IgG > IgM, and 4th group defined by IgG ≈ IgA > IgM

In our cohort, 28 patients had at least three serum samples adequate for evaluation of IgG, IgA, and IgM levels over time. The most prevalent L1-antibody pattern at baseline (found in 13 cases) is characterized by IgG >  > IgA > IgM. The second most prevalent pattern is IgG > IgA > IgM (detected in six cases). Five and four cases show IgA > IgG > IgM and IgG ≈ IgA > IgM, respectively (Fig. 1).

**Fig. 2:**
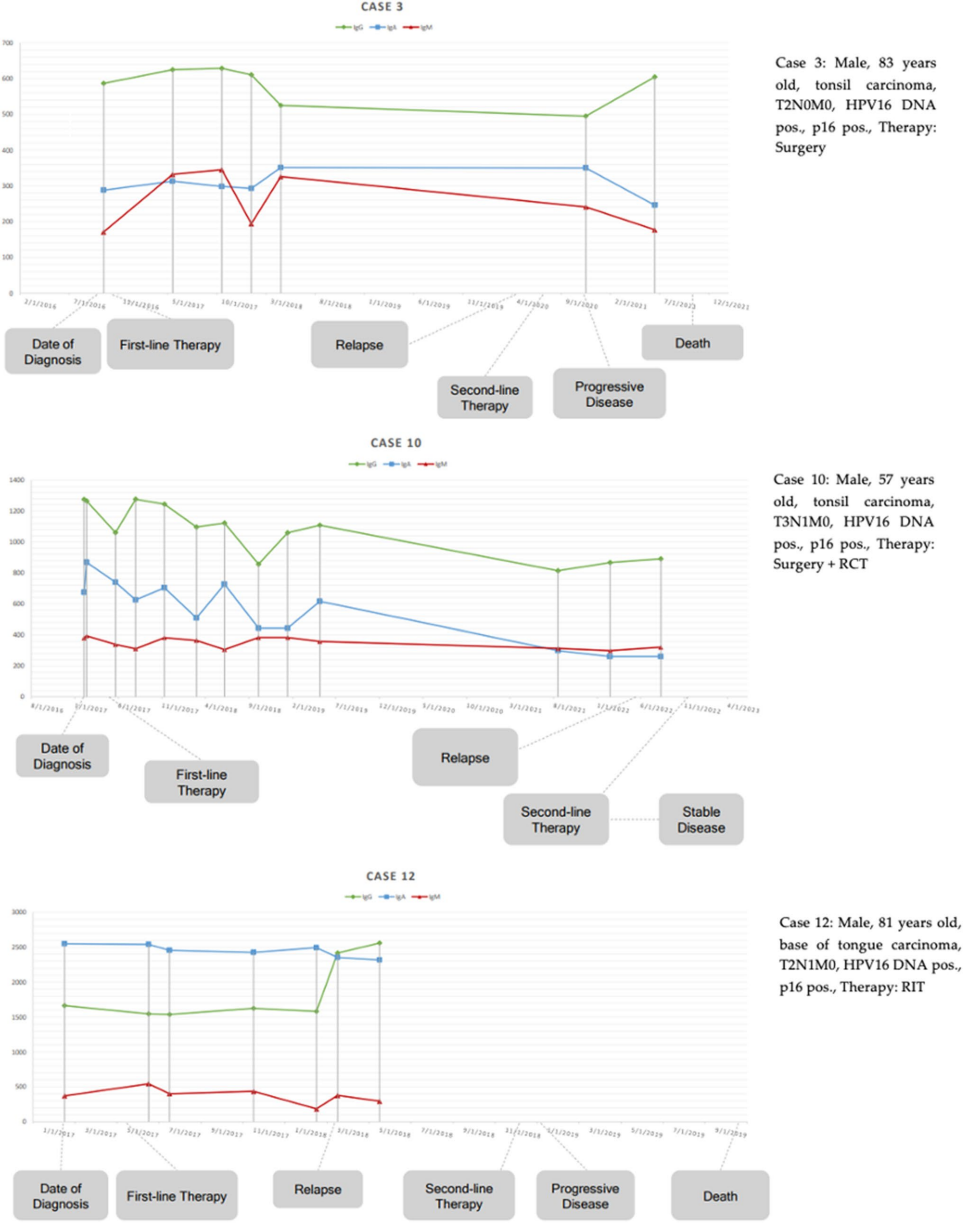
L1-antibody patterns in relapsing disease

There were three patients (cases 3, 10, 12) with recurrent disease following first-line therapy (surgery (case 3), surgery + radiochemotherapy (RCT) (case 10), and radioimmunotherapy (RIT) (case 12)). The time to relapse was between 10 and 65 months. The primary location has been the tonsils (2 cases), or the BOT (1 case). Patients with relapse were male, aged between 57 and 83. All of the relapsed patients had a negative smoking history and denied regular alcohol consumption at diagnosis. HPV16-DNA has been identified in all cases of recurrence. Relapse was not associated with advanced tumor stage (case 3: AJCC I, case 10 and 12: AJCC II). Three patients received second-line therapy (SLT). SLT included immunotherapy (2) or radiotherapy (1). Two out of three patients treated with radiotherapy (RT) or IT developed progressive disease following second-line therapy. See Table 2 for an overview of treatment regimens and therapy success and see Fig. 2 for antibody curves in patients with recurrent disease.

**Table 2:** Overview of treatment regimens and therapy success

First-line therapy		Best response to first-line therapy	Relapse	Second-line therapy		Best response to second-line therapy	
	Number of patients	CR^f^			Number of patients		Number of patients
RT^a^	2	2	0				
RIT^b^	3	3	1	IT^d^	1	PD^g^	1
RCT^c^	9	9	0				
RCT + RIT	1	1	0				
Surgery	3	3	1	RT	1	PD	1
Surgery + RCT	10	9	1	IT	1	SD^i^	1
Surgery + RT	6	6	0				

^a^Radiotherapy

^b^Radioimmunotherapy

^c^Radiochemotherapy

^d^Immunotherapy

^e^No treatment

^f^Complete remission

^g^Progressive disease

^h^Lost to follow-up

^i^Stable disease

A detailed assessment of potential risk factors for disease recurrence reveals that two of the three patients were over 80 years old. In patient number 3 (83 years old), adjuvant therapy was foregone due to his old age after R0 tumor resection. Four years later, a local recurrence ipsilaterally in the epipharynx was diagnosed. Patient number 12 (81 years old) initially received radiotherapy with consecutive cetuximab therapy, which was discontinued after 4 cycles due to a severe skin reaction. One year later, lung metastases were diagnosed. Patient number 10 showed extensive lymph node metastasis with infiltration of the internal jugular vein intraoperatively. Despite adjuvant radiochemotherapy, a metastasis in the area of the ipsilateral scalene muscles and multiple bone metastases were diagnosed 5 years later.

**Fig. 3:**
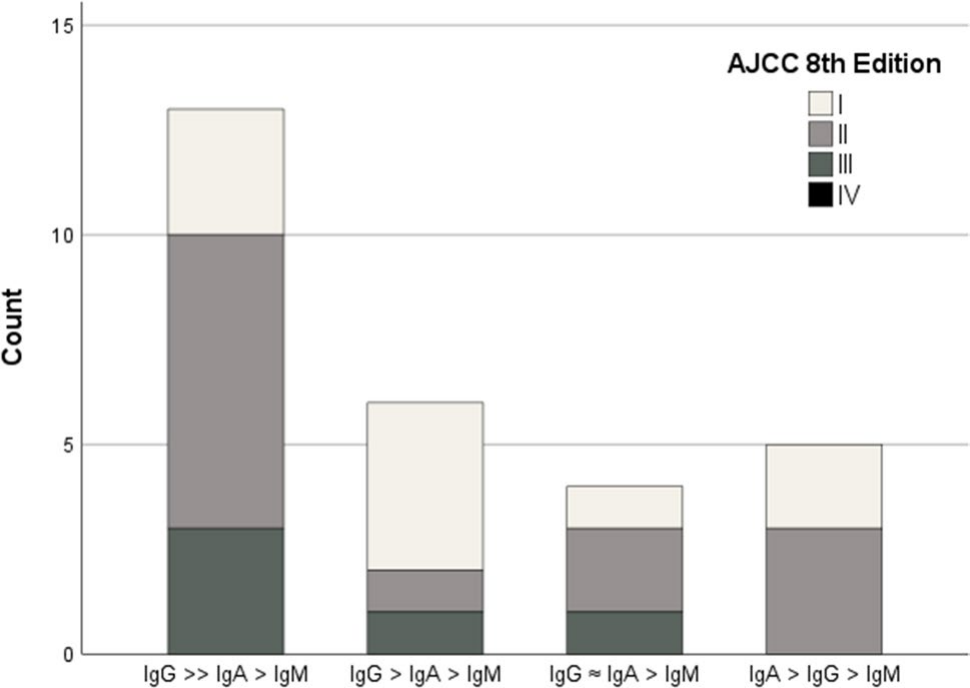
Histogram showing distribution of AJCC stages (8th Edition) in antibody subclass patterns at diagnosis. Patterns are defined by: 1st IgG >  > IgA > IgM, 2nd IgG > IgA > IgM, 3rd IgG ≈ IgA > IgM, and 4th IgA > IgG > IgM

Relapse was associated with an increase of HPVL1-IgG in all cases. In cases 10 and 12, L1-IgG increased four, and two months prior to relapse, respectively. Case three did present a similar, but not equal pattern, due to a delay of seven months between relapse and consecutive sampling. At initial diagnosis, we found IgA over IgG levels in case 12, switching to IgG over IgA several months before relapse was diagnosed. Case 12 had higher IgA levels compared to cases three and 10. We were not able to identify significant differences regarding IgM levels in cases 10 and 12. IgM peaked approx. two months following surgery in case three and consecutively declined over the following four years (Fig. 2). Although Chi^2^-Test showed no statistically significant (*p* > 0.05) difference of antibody patterns between AJCC-stages (*p* = 0.261), we found that the more dominant the IgG level at diagnosis is, the more prevalent are higher AJCC-stages (Fig. 3).

IgG/IgA-Ratio at diagnosis did not significantly differ between AJCC-subgroups (Kruskal–Wallis-*H*-Test *p* = 0.326). The Mann–Whitney *U* Test showed no statistically significant difference regarding IgG/IgA ratio at diagnosis and gender (*p* = 0.853), smoking-status (*p* = 0.869), alcohol-status (*p* = 0.059), and relapse versus no relapse (*p* = 0.511). Supp. 2 shows IgG/IgA ratio at diagnosis (T0), at treatment start (T1), 3–6 months post treatment (T2), and 6–12 months post treatment (T3). The Mann Whitney U Test showed no significant difference at T0 (*p* = 0.547), at T1 (*p* = 0.427), at T2 (*p* = 0.622), and at T3 (*p* = 0.484) between the relapse and the no relapse cohort.

## Discussion

We conducted a study to analyze the distribution of post-treatment HPV L1-AB subclasses in HPV-positive OPSCC in the course of therapy and follow-up. To our knowledge, this is the first study in which AB-kinetics of L1-IgA, -IgM, and -IgG are investigated over time.

Although HPV-positive OPSCC is associated with a favorable prognosis, up to 25% of patients develop relapsing disease within five years after first-line treatment [16]. Hence robust and easy-to-use clinical monitoring is needed to effectively evaluate post-treatment disease status. In the past few years different serological tests have been studied in HPV-positive OPSCC. Antibody-based serological tests revealed that E6 and E7 oncoproteins of high-risk HPVs strongly correlate to HPV-driven malignancies, whereas L1-antibodies are regarded as cumulative-exposure markers [17, 18]. Oton-Gonzales et al. used a commercial kit (HPV16 L1, Cusabio, Houston, TX, USA) to investigate serological L1-IgG kinetics in 20 HPV-positive HNSCC cases [17]. They found that L1-IgG antibody levels did not significantly vary during a follow-up period of 24 months (*p* > 0·05) [17]. Piontek and co-workers conducted a study in 184 HPV16-driven invasive cervical cancer patients to characterize post-treatment AB-dynamics using multiplex HPV serological testing (GST-derived antigens) [18]. They showed that L1 antibody levels are stable over time whereas E6 and E7 AB levels decreased after cervical cancer treatment [18]. Both studies confirm the hypothesis that L1 IgG-ABs remain stable over time. In contrast to available data, findings of our previously published paper suggest that HPV16-L1 DRH1 epitope-specific antibodies are linked to HPV16-induced tumor recurrence [14]. In the current study four different patterns of antibody subclasses were identified at diagnosis in the following frequency: 1. IgG >  > IgA > IgM, 2. IgG > IgA > IgM, 3. IgA > IgG > IgM, and 4. IgG ≈ IgA > IgM. Although not statistically significant (*p* = 0.326), higher IgG/IgA-Ratios at diagnosis gravitated towards higher AJCC-stages. These findings indicate that the dominance of IgG at the time of diagnosis might play a role in further risk-stratification of HPV-positive OPSCCs. Relapse was in three of three cases associated with an increase of HPV-L1-IgG beforehand, which, on the other hand, would support the conclusion of our previous study that HPV-L1 antibody detection might be used as a post-treatment biomarker [14, 15]

**Fig. 4:**
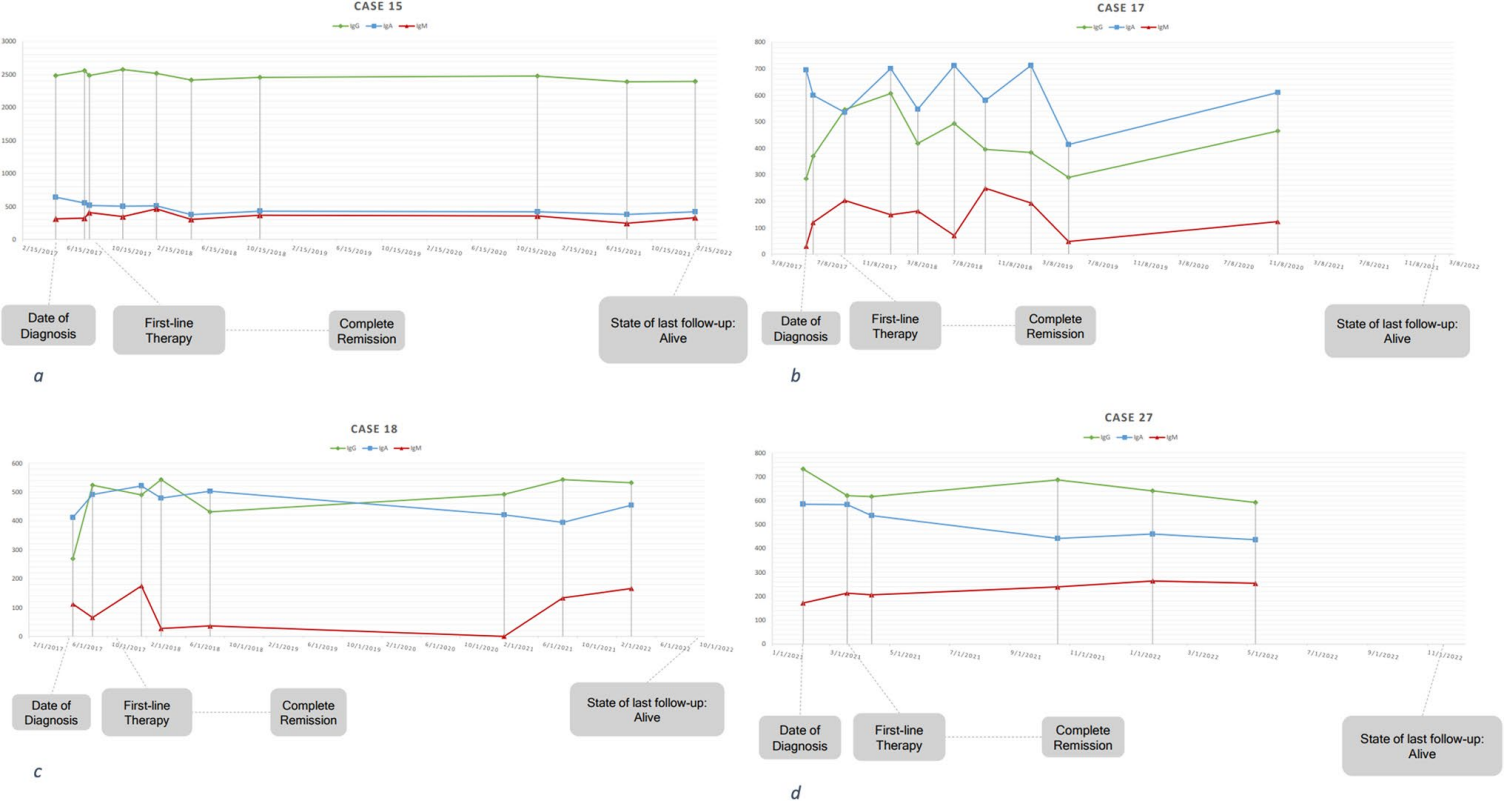
Excerpts of individual L1-antibody patterns. **a** Case 15: male, 57 years old, tonsil carcinoma, T2N1M0, HPV16 DNA pos., p16 pos., Therapy: RCT. **b** Case 17: male, 67 years old, tonsil carcinoma, T2N0M0, HPV16 DNA pos., p16 pos., Therapy: surgery + RT. **c** Case 18: male, 77 years old, tonsil carcinoma, HPV16 DNA pos., p16 pos., Therapy: RIT. **d** Case 27: female, 61 years old, tonsil carcinoma, HPV16 DNA pos., p16 pos., Therapy: surgery + RCT

Although analyses of the entire cohort did not show statistically significant AB-fluctuation over time (mainly due to the small sample size), individual antibody profiles revealed some interesting abnormalities (Fig. 4).

Serum IgA dominance at diagnosis was observed in 50% (2/4) cases primary located at the BOT. The remaining two cases presented with IgG dominance but subsequently switched to IgA dominance over time. IgA dominance at diagnosis in cases primary located at the tonsillar complex, has been found in 16.6% (3/18) cases. In one case IgA and IgG antibodies reached an equal level over time. IgA dominance at diagnosis was associated with a superior disease-free survival. Only one out of 7 cases developed relapse, associated with an IgA to IgG switch several months prior to clinical diagnosis of tumor recurrence. We observed an IgA to IgG switch in one additional case, but so far without any hint of disease recurrence. IgG dominance at the time of tumor recurrence has been found in all three clinically confirmed recurrences. So far there is no reasonable explanation for the association between superior clinical outcome and serum IgA dominance over time. Literature supports the assumption that IgA is the predominant AB-subclass found in saliva, and IgG in serum [19]. IgM to IgA AB-class switch in naive B-cells seems to be predominantly controlled by TGF-β [20]. HPV E6/E7 oncoproteins can upregulate TGF-β promotor activity resulting in TGF-β overexpression, which stimulates survival and proliferation of cervical cancer cell lines. Interestingly, TGF-β overexpression has also been described in OPSCC [21]. It is unclear whether OPSCC-cells themselves or cells of the tumor microenvironment are responsible for the expression of different amounts of TGF-β which may explain IgA or IgG dominance. Von Witzleben and colleagues found that HPV E2 and E7 related IgA antibodies have not been associated with a superior overall survival in HPV-positive OPSCC patients [22], which may be indicative that the observed effect could rather be related to the HPV late proteins. A reason for this difference could be the high molecular weight of L1 based virus like particles that in contrast to the very small early proteins are able to bind to toll like receptors (TLR4) of the innate immune system, possibly thereby changing the cytokine network of the tumor microenvironment which in turn may influence the adaptive immune response as well [23].

## Conclusion

Despite the limited number of cases, our prospective pilot study to detect HPV-L1 antibody subclasses showed interesting trends that may provide additional utility for risk stratification at diagnosis and for monitoring possible recurrent disease in HPV-positive OPSCC patients. Of course, prospective studies with larger sample sizes and longer follow-up are necessary to confirm these assumptions.

### Supplementary Information

Below is the link to the electronic supplementary material.

Supplementary file1 Supp. 1 Serum sampling algorithm. n = Number of serum samples collected per treatment period. 28 patients with at least three serum samples were included in the analysis. (PPTX 47 KB)

Supplementary file2 Supp. 2 Boxplot IgG/IgA ratio at four separate timepoints. IgG/IgA ratio at T0 = at diagnosis, T1 = treatment start, T2 = first follow-up 3-6 months post treatment, and T3 = second follow-up 6-12 months post treatment for relapse and no relapse cohort. Boxes represent 1st quartile, median, and the 3rd quartile. Upper whiskers represent 1.5 times of the 3rd quartile, lower whiskers represent the 1.5 times of the 1st quartile. (PPTX 58 KB)

## Data Availability

All data generated or analyzed during this study are included in this published article.
