# Supplementary material for: Detection of antibody subclasses IgA, IgM and IgG against HPV L1 in HPV-positive oropharyngeal squamous cell carcinoma patients: a pilot study
Source: Eur Arch Otorhinolaryngol. 2024 Mar 5;281(5):2637–44. doi: 10.1007/s00405-024-08537-9 (PMC11023979; doi:10.1007/s00405-024-08537-9)
Supplement: Supplementary file 1 — Supplementary file1 Supp. 1 Serum sampling algorithm. n = Number of serum samples collected per treatment period. 28 patients with at least three serum samples were included in the analysis. (PPTX 47 KB) [file 405_2024_8537_MOESM1_ESM.pptx]

## Slide 1
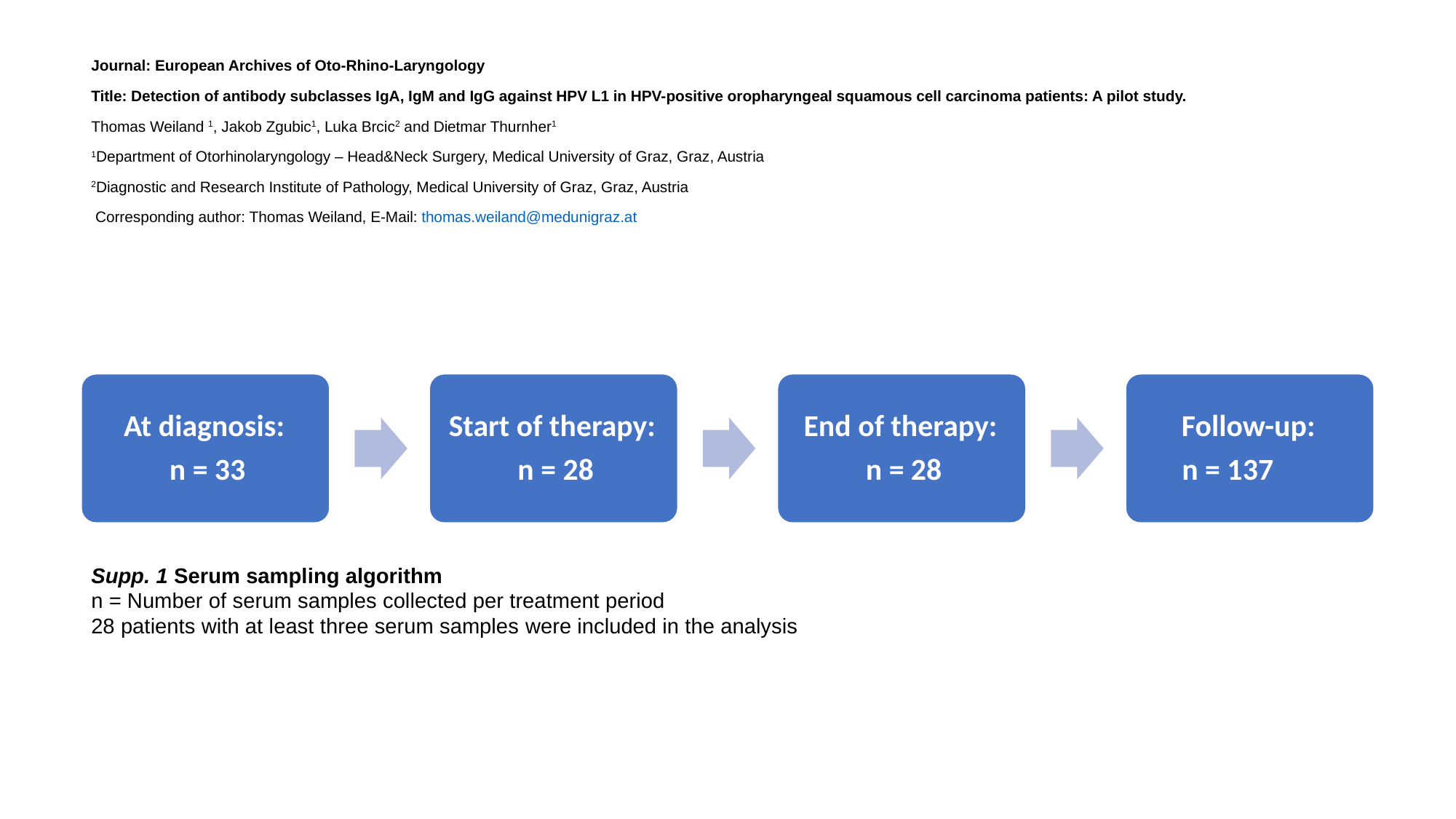

Journal: European Archives of Oto-Rhino-Laryngology
Title: Detection of antibody subclasses IgA, IgM and IgG against HPV L1 in HPV-positive oropharyngeal squamous cell carcinoma patients: A pilot study.
Thomas Weiland 1, Jakob Zgubic1, Luka Brcic2 and Dietmar Thurnher1
1Department of Otorhinolaryngology – Head&Neck Surgery, Medical University of Graz, Graz, Austria
2Diagnostic and Research Institute of Pathology, Medical University of Graz, Graz, Austria
 Corresponding author: Thomas Weiland, E-Mail: thomas.weiland@medunigraz.at
Supp. 1 Serum sampling algorithm
n = Number of serum samples collected per treatment period
28 patients with at least three serum samples were included in the analysis
