# Supplementary material for: Detection of antibody subclasses IgA, IgM and IgG against HPV L1 in HPV-positive oropharyngeal squamous cell carcinoma patients: a pilot study
Source: Eur Arch Otorhinolaryngol. 2024 Mar 5;281(5):2637–44. doi: 10.1007/s00405-024-08537-9 (PMC11023979; doi:10.1007/s00405-024-08537-9)
Supplement: Supplementary file 2 — Supplementary file2 Supp. 2 Boxplot IgG/IgA ratio at four separate timepoints. IgG/IgA ratio at T0 = at diagnosis, T1 = treatment start, T2 = first follow-up 3-6 months post treatment, and T3 = second follow-up 6-12 months post treatment for relapse and no relapse cohort. Boxes represent 1st quartile, median, and the 3rd quartile. Upper whiskers represent 1.5 times of the 3rd quartile, lower whiskers represent the 1.5 times of the 1st quartile. (PPTX 58 KB) [file 405_2024_8537_MOESM2_ESM.pptx]

## Slide 1
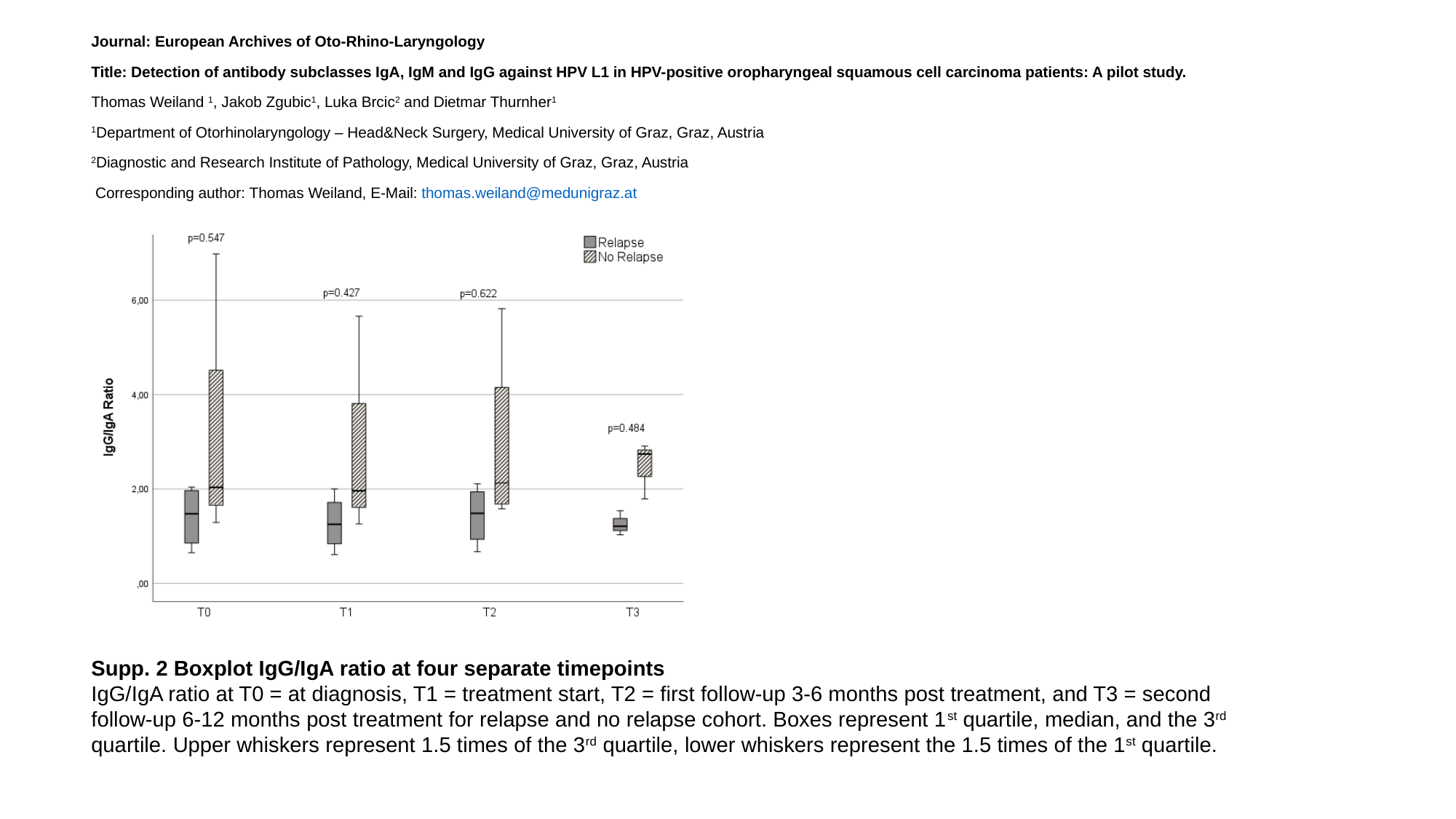

Journal: European Archives of Oto-Rhino-Laryngology
Title: Detection of antibody subclasses IgA, IgM and IgG against HPV L1 in HPV-positive oropharyngeal squamous cell carcinoma patients: A pilot study.
Thomas Weiland 1, Jakob Zgubic1, Luka Brcic2 and Dietmar Thurnher1
1Department of Otorhinolaryngology – Head&Neck Surgery, Medical University of Graz, Graz, Austria
2Diagnostic and Research Institute of Pathology, Medical University of Graz, Graz, Austria
 Corresponding author: Thomas Weiland, E-Mail: thomas.weiland@medunigraz.at
Supp. 2 Boxplot IgG/IgA ratio at four separate timepoints
IgG/IgA ratio at T0 = at diagnosis, T1 = treatment start, T2 = first follow-up 3-6 months post treatment, and T3 = second follow-up 6-12 months post treatment for relapse and no relapse cohort. Boxes represent 1st quartile, median, and the 3rd quartile. Upper whiskers represent 1.5 times of the 3rd quartile, lower whiskers represent the 1.5 times of the 1st quartile.
